# Conservative Treatment for Cystic Duct Stenosis in a Child

**DOI:** 10.1155/2013/146261

**Published:** 2013-01-20

**Authors:** Marco Gasparetto, Laura Giordano, Mara Cananzi, Valeria Beltrame, Gianni Bisogno, Graziella Guariso

**Affiliations:** ^1^Unit for Gastroenterology, Digestive Endoscopy, Hepatology, and Care of Children with Liver Transplants, Department of Women's and Children's Health, Padova University Hospital, Via Giustiniani 3, 35128 Padova, Italy; ^2^Department of Medical Diagnostic Sciences and Special Therapies, Radiology Institute, Padova University Hospital, Via Giustiniani 1, 35128 Padova, Italy; ^3^Hematology and Oncology Unit, SPeLeS Project Coordinator, Department of Women's and Children's Health, Padova University Hospital, Via Giustiniani 3, 35128 Padova, Italy

## Abstract

*Introduction*. Few cases of common bile duct stenosis have been reported in the literature, and observations of strictures in the cystic duct are even more rare. Surgical cholecystectomy is the treatment needed in most cases of gallbladder hydrops. This paper describes the diagnosis and successful medical treatment of a rare pediatric case of cystic duct stenosis and gallbladder hydrops. *Case Report*. A formerly healthy one-year-old girl was admitted with colicky abdominal pain. Blood tests were normal, except for an increase in transaminases. Abdominal ultrasound excluded intestinal intussusception and identified a distended gallbladder with biliary sludge. MR cholangiography revealed a dilated gallbladder containing bile sediment and no detectable cystic duct, while the rest of the intra- and extrahepatic biliary tree and hepatic parenchyma were normal. This evidence was consistent with gallbladder hydrops associated with cystic duct stenosis. The baby was treated with i.v. hydration, corticosteroids, antibiotics, and ursodeoxycholic acid. Her general condition rapidly improved, with no further episodes of abdominal pain and normalization of liver enzymes. This allowed to avoid cholecystectomy, and the child is well 1.5 years after diagnosis. *Conclusions*. Although cholecystectomy is usually necessary in case of gallbladder hydrops, our experience suggests that surgical procedures can be avoided when the distension is caused by a cystic duct stenosis.

## 1. Introduction

Acute gallbladder distension (hydrops) is unusual in pediatric age [1, 2]. It may be a consequence of obstruction of the cystic duct caused by gallstones, cholangitis, sclerosing cholangitis, cystic fibrosis [3], congenital or postoperative biliary malformations [4], or benign or malignant lesions [5, 6]. Laparoscopic cholecystectomy is usually warranted [7–10]. There are few reports of stenosis of the common bile duct in the literature, and the observations of strictures located in the cystic duct are even rarer. This paper describes and discusses the diagnostic and therapeutic workup in a child with stenosis of the cystic duct and gallbladder hydrops.

## 2. Case Report

**Figure 1:**
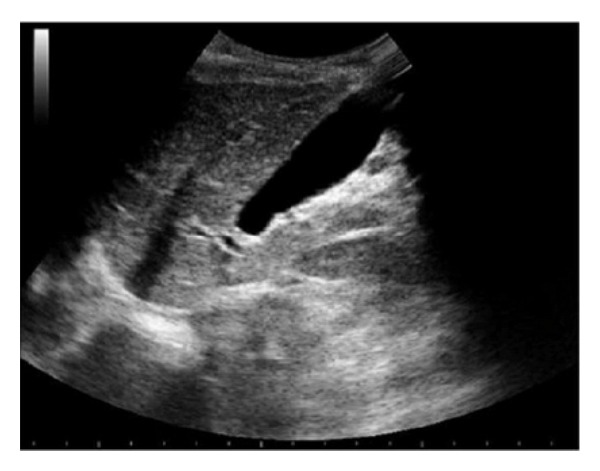
Hepatic ultrasound showing a distended gallbladder.

**Figure 2:**
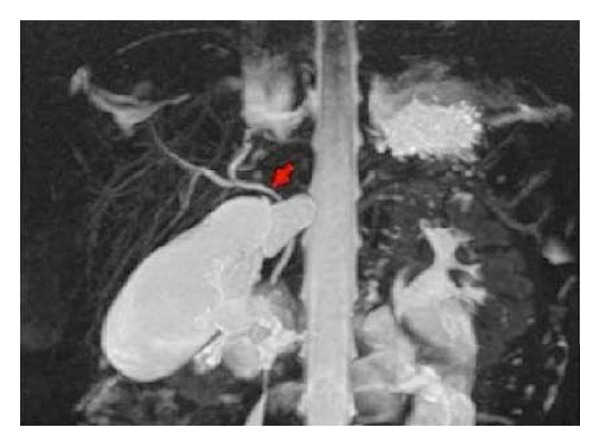
MR cholangiography: a coronal maximum intensity projection (MIP) shows a distended gallbladder with no sign of the cystic duct (arrow).

A formerly healthy one-year-old Caucasian girl was admitted with colicky abdominal pain and a history of two episodes of vomiting on the previous day, and a bout of gastroenteritis during the week before, with isonatremic dehydration. On admission, the child's general condition seemed poor. Her abdomen was distended and diffusely painful, particularly in the middle right quadrant, where a soft mass was detectable. Blood tests were normal, except for an increase in liver enzymes (ALT 101 U/L). Abdominal ultrasound (US) showed no intestinal intussusception, but a severely distended gallbladder (7.5 cm in diameter) containing biliary sludge (Figure 1). MR cholangiography (Figure 2) confirmed the gallbladder distension, while the cystic duct was not detectable; the rest of the intra- and extrahepatic biliary tree and the hepatic parenchyma were normal.

Malformative and infectious/inflammatory hypotheses were considered, as well as the possibility of bile concentration and thickening secondary to severe, acute dehydration.

The results of blood tests to rule out the main hematological (complete blood count, reticulocytes, indices of hemolysis, peripheral smear), hepatic (indices of cholestasis, pancreatic amylases, lipid profile), and infectious (inflammation indexes, viral and bacterial detection on blood and feces) causes of cystic duct stenosis were all normal.

No stones were detected, so the picture was consistent with gallbladder hydrops associated with a stricture of the cystic duct resulting from a congenital malformation.

The child showed signs of spontaneous clinical improvement, so cholecystectomy was withheld and the child was treated with i.v. hydration, corticosteroids, antibiotics, and ursodeoxycholic acid. She continued to improve, with a concomitant decrease in her transaminases and the dimensions of her gallbladder. The girl has since been followed up routinely for 1.5 years and has so far shown a regular growth, with no more episodes of abdominal pain. A mass is still detectable 2-3 cm from the right mid-costal arch, and hepatic US still reveals a distended gallbladder with an elongated shape (5 cm), thickened walls, and signs of biliary sludge (mainly in the infundibulum). Blood tests, including transaminases, are all normal.

## 3. Discussion

Acute hydrops of the gallbladder in pediatric patients is often secondary to systemic illness (e.g., infections) [2]. Various pathogenic mechanisms have been suggested, including cystic duct obstructions, with subsequent bile concentration and stasis causing ineffective gallbladder emptying. Acute gallbladder hydrops has also been described in association with Kawasaki syndrome, as well as with staphylococcal or streptococcal upper respiratory tract infections, with associated toxin production [2]. Other infections (Leptospirosis, Pseudomonas sepsis, Epstein-Barr viral infection, viral hepatitis, Salmonella Enteric fever, and Cryptosporidium infections [11]) have also, less frequently, been associated with gallbladder hydrops. A few cases have been reported in infants and children with Sjogren's syndrome, Henoch-Shonlein purpura, Wilson's disease, and hypokalemia secondary to Bartter's syndrome [2].

In our case, infectious causes were ruled out and repeated radiological investigations were consistent with a congenital malformation causing stenosis of the cystic duct. To our knowledge, only one previous pediatric case of isolated cystic duct stricture has been reported as yet [12], in a patient who was treated with ursodeoxycholic acid, antibiotics, and steroids.

Our experience confirms that congenital cystic duct stenosis can be treated conservatively. Supportive medical treatment has so far been effective in helping the child to recover from her acute symptoms without resorting to cholecystectomy. In particular, the administration of corticosteroid treatment was decided with a view to reduce any inflammatory component of the stricture that might be secondary to the congenital malformation and bile stasis. After a relatively long followup (1.5 years), we cannot rule out the possibility of our case needing surgery in the future, but for the time being the child has been able to grow normally and conduct a normal life.

## 4. Conclusion

Our experience supports the adoption of a conservative approach in children with gallbladder hydrops and congenital cystic duct stenosis. A careful followup is recommended to establish if and when any surgery is needed.
